# Weakening the nuclear envelope: Lamin B receptor in melanoma metastasis

**DOI:** 10.1002/1878-0261.70281

**Published:** 2026-06-07

**Authors:** Francesca Lorenzini, Maria Caterina Mione

**Affiliations:** ^1^ Experimental Cancer Biology, Department of Cellular, Computational and Integrative Biology (CIBIO) University of Trento Italy

**Keywords:** cancer invasion, confined migration, Lamin B receptor (LBR), metastatic melanoma, nuclear envelope fragility

## Abstract

Nuclear envelope fragility has been identified as a hallmark of the metastatic process. Indeed, cancer cells undertake a challenging journey through diverse environments during metastasis, notably squeezing through narrow spaces and dense collagen matrices. In their report, Baird et al. identify a key player in nuclear fragility in confined cancer cell migration: the Lamin B receptor. Its overexpression and clustering in specific regions of the nuclear envelope increase nuclear distortion and fragility, rendering nuclei more susceptible to rupture with consequent chromatin extrusion and genomic instability. Elucidating the mechanisms underlying this process, particularly the receptor's sterol reductase activity, represents a promising step towards the identification of novel therapeutic targets.

## Graphical abstract

LBR‐driven nuclear fragility supports melanoma invasion. A: Melanocyte presents low LBR (Lamin B Receptor) levels, maintaining nuclear integrity and lamina‐chromatin tethering. B: During malignant progression, upregulation of LBR clusters at the INM (Inner Nuclear Membrane) during confined migration causes local lamina weakening and cholesterol‐mediated membrane fluidity. C: Mechanical pressure triggers NE (Nuclear Envelope) blebbing, rupture, and DNA damage, fuelling genomic instability and enhanced metastatic invasion. By reducing cholesterol level, statin may decrease tumour invasiveness.

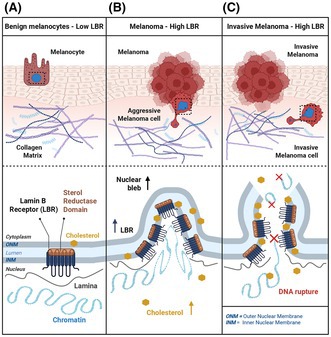

**Abbreviations**
INM: inner nuclear membrane
KD: knockdown
LBR: Lamin B receptor
NE: nuclear envelope
ONM: outer nuclear membrane
RS domain: arginine (R)/serine (S)‐rich domain

## 1 Introduction

Metastatic spread is a complex, multistep process leading to the formation of secondary tumours. While the individual stages of this cascade have been identified, the precise molecular mechanisms underlying metastasis remain to be fully elucidated. Understanding these processes is fundamental, as metastatic dissemination is the primary cause of cancer‐related mortality [1]; in the case of skin cancer, melanoma represents the leading cause of death due to its aggressive nature [2].

During the metastatic cascade, melanoma cells navigate diverse microenvironments characterized by varying degrees of pressure and stiffness. To adapt, cancer cells undergo significant transcriptional reprogramming that translates into structural and metabolic alterations. Primarily, melanoma cells must transition from a proliferative state to a migratory and invasive phenotype, a process generally known as phenotype switching [3], necessitating a profound internal reorganization. As they move towards distant organs, cells must traverse dense tissues and overcome physical constraints, such as sneaking in dense dermal tissue and squeezing through a stiff vascular wall during intravasation, extravasation and trans endothelial migration.

While the cytoplasm is relatively flexible, the nucleus possesses distinct mechanical properties that make it stiffer and more resistant to deformation [4]. Nuclear mechanics are governed by a complex interplay between the cytoskeleton, integral nuclear envelope (NE) proteins, the lamin nucleoskeleton (lamina) and chromatin. Mechanical forces during metastasis can lead to NE rupture, resulting in the exposure of the genome to cytoplasmic damaging agents and the mislocalization of DNA repair factors, which in turn promotes heritable DNA damage and chromothripsis [5, 6]. Typically, NE damage and ruptures occur at ‘NE blebs’, which are protrusions where intranuclear pressure causes the outflow of nucleoplasm and, occasionally, chromatin [5].

The NE consists of the outer nuclear membrane (ONM) and the inner nuclear membrane (INM). The INM is enriched with integral membrane proteins that form bridges with the lamina and heterochromatin, thereby organizing the genome [4]. In this context, as metastatic melanoma exhibits a high mutational burden, it is hypothesized that genomic heterogeneity is supported by this inherent NE fragility. In the highlighted work, the authors combine high‐resolution imaging and molecular biology with organoid and *in vivo* modelling to investigate how NE fragility drives increased invasiveness and identify the sterol reductase activity of the Lamin B receptor (LBR) as a key mediator of this process [7].

## 2 Lamin B receptor drives nuclear envelope instability in melanoma

Analysis of altered nuclear envelope (NE) gene expression across 10 cancer subtypes revealed that transcriptional dysregulation of NE components is a common hallmark of malignancy. This dysregulation is closely linked to increased NE fragility under mechanical stress, an intrinsic property observed in melanoma, breast, and prostate cancer cell lines. By analysing human melanoma samples at various stages of progression, the authors demonstrated that alterations in nuclear morphology and NE architecture correlate with malignant transformation, highlighting the fundamental role of these transcriptional changes in disease progression.

Bioinformatic analysis of human melanoma samples showed that nearly all nuclear‐associated genes are upregulated in metastatic melanoma compared to benign nevi. To pinpoint key drivers, the authors performed a siRNA knockdown (KD) screen of the top 30 upregulated NE genes in a malignant melanoma cell line (1205Lu). This led to the identification of the Lamin B Receptor (LBR) as a primary mediator of NE rupture. LBR is an integral INM protein featuring a TUDOR and RS domain for binding lamina and heterochromatin, alongside a C‐terminal transmembrane domain with sterol reductase activity involved in cholesterol biosynthesis. Notably, LBR overexpression in immortalized normal melanocytes was sufficient to induce NE fragility, mimicking the phenotype of metastatic cells.

## 3 LBR couples cholesterol metabolism to nuclear envelope fragility

Mechanistic investigations using immunofluorescence revealed that LBR does not maintain overall NE architecture, as LBR‐KD did not alter levels of other structural proteins and nuclear pores. Instead, LBR regulates the mechanical contiguity of the INM and ONM. In confined melanoma cells, LBR clusters in specific INM regions, promoting local lamina breaks and INM weakening. This allows mechanical pressure to drive chromatin extrusion into NE blebs, eventually leading to ONM rupture and chromatin spillage.

To further dissect these functions, the authors employed a mutant add‐back approach. They demonstrated that NE fragility and nuclear stiffness are governed by distinct domains of LBR: the RS domain regulates nuclear deformability, while the C‐terminal sterol reductase activity promotes NE fragility. Specifically, LBR upregulation increases cellular cholesterol levels during confinement. Pharmacological depletion of cholesterol using methyl‐β‐cyclodextrin or simvastatin rendered nuclei more deformable and fluid‐like, confirming that LBR‐mediated cholesterol metabolism dictates NE mechanical stability.

Finally, the authors validated these findings in complex models, including tumour organoids embedded in collagen matrices and xenograft mouse models. These experiments confirmed that LBR upregulation facilitates NE rupture and significantly enhances cell invasion within a confining extracellular matrix (ECM). Collectively, these results establish increased expression of LBR in melanoma as a key molecular switch that modulates nuclear mechanics and fuels the metastatic process.

## 4 Key findings

In this seminal work [7], the authors elucidate how an intrinsic transcriptional event, such as the upregulation of a single gene, profoundly alters the mechanical properties of the nucleus, thereby driving the acquired aggressiveness of melanoma cells. Through a combination of meticulous molecular biology techniques and high‐resolution imaging, the researchers successfully characterized the precise mechanisms underlying NE fragility.

A central discovery of the study is the identification of LBR clusterization as the primary driver of NE blebbing and subsequent rupture. The authors demonstrate that in metastatic cells, LBR does not remain uniformly distributed; instead, it aggregates into high‐density clusters within specific regions of the inner nuclear membrane. These clusters act as focal points of structural weakness, where the local integrity of the nuclear lamina is compromised, facilitating the formation of nuclear blebs under mechanical pressure.

The study further sheds light on the interplay between transcriptional regulation, metabolism and mechanics. By investigating the sterol reductase activity of LBR, the authors established a direct link between cellular cholesterol metabolism and the physical phenotype of the nucleus. They found that LBR clusterization is intrinsically tied to its enzymatic role, suggesting that localized metabolic shifts at the nuclear membrane dictate the deformability and fragility of the nucleus.

Ultimately, this research connects the dots between a genetic ‘trigger’ and a physical ‘outcome’, showing how melanoma cells exploit metabolic and structural pathways to survive the extreme physical constraints and gain advantages for the metastatic journey. This provides a new framework for understanding how cancer cells harness nuclear instability to foster genomic heterogeneity and invasive potential.

## 5 Outlook

This work elucidates a sophisticated mechanism by which cancer cell migration is sustained despite extreme deformation of the cytoplasm and its nucleus, and that ultimately leads to disease progression and increased aggressiveness. The observed dynamics of the nucleus during migration highlights that functional NE and DNA damage repair systems are indispensable for cancer cell survival [5]. In this context, exploring strategies that further exacerbate NE fragility could provide an attractive opportunity to induce synthetic lethality of melanoma cells engaging in metastatic migration supported by the increase of LBR expression. Indeed, by deliberately overwhelming the adaptive mechanisms that cancer cells rely on to survive the stress of migration and nuclear squeezing, it may be possible to exploit this novel therapeutic vulnerability to limit the metastatic process.

Furthermore, this research demonstrates that melanoma migration and invasiveness are driven not only by external signals from the tumour microenvironment but also by intrinsic changes, like changes in gene expression, that dictate the fate of tumour progression. This paradigm is supported by recent findings, such as those by Hunter et al. [8], which show that chromatin remodelling mediated by the upregulation of the DNA‐binding protein HMGB2 promotes phenotype switching in confined cells. While LBR is a central figure in this study, other INM proteins, such as lamina‐associated polypeptide 1 (LAP1), have recently been identified as master regulators of NE remodelling during melanoma progression [9]. Together, these data reveal a complex regulatory network that integrates cytoskeletal dynamics and metabolic shifts to facilitate tumour progression. A particularly striking aspect of this work is the involvement of sterol metabolism in NE fragility. The role of cholesterol in directly influencing the tumour's ability to gain a competitive advantage during migration is an emerging field of interest. Different studies are already investigating the efficacy of repurposing statins to inhibit tumour proliferation and migration [10]. Statins, by inhibiting cholesterol synthesis, may act through a dual mechanism: suppressing oncogenic signalling pathways [11] and providing structural stability by reducing NE fragility.

Despite these promising insights, it is important to note that much of the mechanistic detail in this study was derived from a single cell line. To strengthen these findings and ensure their clinical relevance, it is essential to validate this LBR‐mediated mechanism across a broader panel of melanoma cell lines and patient‐derived models. Furthermore, determining whether these results can be translated to other cancer types, such as breast or prostate cancer, which also are characterized by a restrictive microenvironment, will be crucial to establish LBR as a universal therapeutic target in oncology.

## Conflict of interest

The authors declare no conflict of interest.

## Author contributions

FL wrote the manuscript, MCM reviewed it.
